# Trisomy 18 (Edwards syndrome) and major gastrointestinal malformations

**DOI:** 10.1590/S1516-31802013000100025

**Published:** 2013-04-01

**Authors:** Rafael Fabiano Machado Rosa, Rosana Cardoso Manique Rosa, Marina Boff Lorenzen, Paulo Ricardo Gazzola Zen, Ceres Andréia Vieira de Oliveira, Carla Graziadio, Giorgio Adriano Paskulin

**Affiliations:** I PhD. Clinical Geneticist, Universidade Federal de Ciências da Saúde de Porto Alegre (UFCSPA) and Complexo Hospitalar Santa Casa de Porto Alegre (CHSCPA), Porto Alegre, Rio Grande do Sul, Brazil.; II MD. Postgraduate, Postgraduate Program on Pathology, Universidade Federal de Ciências da Saúde de Porto Alegre (UFCSPA), Porto Alegre, Rio Grande do Sul, Brazil.; III Undergraduate Medical Student, Universidade Federal de Ciências da Saúde de Porto Alegre (UFCSPA), Porto Alegre, Rio Grande do Sul, Brazil.; IV PhD. Adjunct Professor of Clinical Genetics and Professor of the Postgraduate Program on Pathology, Universidade Federal de Ciências da Saúde de Porto Alegre (UFCSPA), and Clinical Geneticist, Universidade Federal de Ciências da Saúde de Porto Alegre (UFCSPA) and Complexo Hospitalar Santa Casa de Porto Alegre (CHSCPA), Porto Alegre, Rio Grande do Sul, Brazil.; V MD. Professor, Instituto de Educação e Pesquisa (IEP), Hospital Moinhos de Vento (HMV), Porto Alegre, Rio Grande do Sul, Brazil.; VI MD. Postgraduate Student, Postgraduate Program on Pathology, and Assistant Professor of Clinical Genetics, Universidade Federal de Ciências da Saúde de Porto Alegre (UFCSPA), and Clinical Geneticist, Universidade Federal de Ciências da Saúde de Porto Alegre (UFCSPA) and Complexo Hospitalar Santa Casa de Porto Alegre (CHSCPA), Porto Alegre, Rio Grande do Sul, Brazil.; VII PhD. Associate Professor of Clinical Genetics and Coordinator of the Postgraduate Program on Pathology, Universidade Federal de Ciências da Saúde de Porto Alegre (UFCSPA), and Clinical Geneticist, Universidade Federal de Ciências da Saúde de Porto Alegre (UFCSPA) and Complexo Hospitalar Santa Casa de Porto Alegre (CHSCPA), Porto Alegre, Rio Grande do Sul, Brazil.

Trisomy 18 or Edwards syndrome is a chromosomal disease characterized by involvement of many organs and systems, and limited survival.^1^ The aim of our study was to determine the frequency and types of major gastrointestinal abnormalities observed among patients with Edwards syndrome. The sample consisted of patients consecutively evaluated over the period between 1975 and 2008 in a clinical genetics service at a referral hospital in southern Brazil. Clinical data and karyotype results were gathered from this hospital’s medical records. We use Fisher’s exact test (two-tailed) to compare frequencies (P values < 0.05 were considered significant). Thus, our sample was composed of 50 patients, of whom 33 (66%) were female. Their ages at the first evaluation ranged from 1 day to 16 years (median 14 days). Eight of the patients (16%) were born in that hospital. 

Presence of a single lineage with free trisomy of chromosome 18 was the main abnormality, observed in 90% of the cases. The remainder consisted of patients with mosaicism. Major gastrointestinal abnormalities were observed in eight patients (16%): two cases of esophageal atresia (4%), three of tracheoesophageal fistula (6%) (one associated with esophageal atresia), one of diaphragmatic hernia (2%) and three of omphalocele (6%). Among these eight patients, five (62%) were female. Their ages at the time of the initial evaluation ranged from one to 70 days (median 7.5 days). Only two patients (25%) presented a clinical suspicion of Edwards syndrome. Additional abnormalities, including minor and major anomalies, were observed in all cases. These included dysmorphic features like a clenched fist with overlapping fingers and major malformations like congenital heart defects. None had a chromosomal constitution with mosaicism. Three patients underwent surgical correction of their defects (one with esophageal atresia, one with tracheoesophageal fistula and one with omphalocele). In all cases, the diagnosis of Edwards syndrome was made only after surgery.

Gastrointestinal malformations are frequent among patients with Edwards syndrome. In the literature, the frequency of esophageal atresia among these patients ranges from 16 to 18%.^2^^,^^3^^,^^4^ Despite the frequency of 4% observed in our study, no statistically significant difference was seen using Fisher’s exact test when we compared it with these studies. However, it is noteworthy that the samples in other studies (n = 24 to 39 patients) were smaller than in ours (n = 50). Patients with esophageal atresia usually present the associated finding of tracheoesophageal fistula,^4^^,^^5^ as observed in one of our two patients. Omphalocele has been described in less than 10% of the patients,^2^^,^^3^^,^^4^^,^^5^ which is compatible with the rate in our study (6%). Despite the possible association described in the literature,^5^ none of our patients with omphalocele presented a neural tube defect. Diaphragmatic hernia is considered to be a less common abnormality, and has been described in less than 8% of the patients.^2^^,^^3^^,^^4^ This frequency was similar to our study (2%). 

Thus, Edwards syndrome should always be considered among dysmorphic children presenting these major gastrointestinal malformations. Diagnosing it is important for proper management and for determining the prognosis for these patients.
